# Ten year experience of pediatric kidney biopsies from a single center in Pakistan

**DOI:** 10.4103/0971-4065.73446

**Published:** 2010-10

**Authors:** A. Absar, M. Diamond, Y. Sonia, R. Arshalooz, A. Safia, K. Waqar, P. Shahid

**Affiliations:** Department of Medicine, Aga Khan University Hospital, Stadium Road, Karachi - 74800, Pakistan

**Keywords:** Kidney, biopsy, pediatric, indications, pathology

## Abstract

There are many established registries of kidney biopsies around the world. In addition, there are several reports available in literature from many countries on pediatric kidney biopsy. This study was done to determine the indications and pathological patterns of kidney biopsies of children referred to our hospital, and compare our data with the data available from other countries. This is a cross-sectional study of pediatric kidney biopsies over a 10-year period, from January 1997 to December 2006. All biopsies were done in Aga Khan University Hospital, Karachi, Pakistan. Age range was from 1 to 14 years. Data were analyzed for indications and histopathological diagnosis. A total of 54 kidney biopsies were included in the initial analysis. Here 13 samples were excluded and final analysis was done on the remaining 41 samples. The most common indication of kidney biopsy was nephrotic syndrome in 25 samples (61%). The most common histopathology was minimal change disease in 15 (37%), followed by focal segmental glomerulosclerosis in 5 (12%) of the biopsies.

## Introduction

A kidney biopsy is relatively uncommon in children compared with adults. A literature review indicates that most of the published reports deal with adult kidney biopsy. This difference is due to the fact that nephrotic syndrome in children is assumed to have minimal disease change, and steroid therapy is instituted first without biopsy.[1,2] Kidney biopsy in nephrotics is done only when they have steroid-resistant or steroid-dependent nephrotic syndrome.[3] Now, with the availability of automated triggered biopsy gun, kidney biopsy is a safe procedure and usually not an issue in decision-making.[4]

There are several published reports on pediatric kidney biopsy from various countries. However, a Medline search revealed that there is no published report from Pakistan on this subject. We did this study to determine the pattern of pathological diagnosis and to identify the indications of renal biopsies in children referred to our institution.

## Materials and Methods

This is a cross-sectional study of pediatric kidney biopsies performed over a period of 10 years between January 1997 and December 2006. All biopsies were done in Aga Khan University Hospital, Karachi, which is a tertiary care medical center located in a large cosmopolitan city of Pakistan. All biopsies performed in Aga Khan University Hospital in children 1-14 years of age were collected.

We excluded biopsies form and there with transplant kidney, tumor, inconclusive results

Minimum workup of the patients included the following: 24-h urine protein or spot urine protein/creatinine ratio; urine microscopy; blood urea nitrogen and serum creatinine; and renal sonogram. Additional tests were performed when indicated.

All biopsies were done by a trained nephrologist or an invasive radiologist. Either the automated biopsy gun or a traditional tru-cut biopsy needle was used. Histopathological evaluation of the biopsy specimens was done by light microscopy and immunofluorescence. Electron microscopy was not available. Biopsies were stained with hematoxylin-eosin and periodic acid schiff. Immunoflourescence staining was done with antibodies against IgG, IgM, IgA, C_3_, and other markers if indicated. All specimens were reviewed by two qualified pathologists. The data were stored and analyzed by statistical package for social sciences (SPSS) version 16.

## Results

Total 54 kidney biopsies were included in the initial analysis. After excluding 13 biopsy samples, there were 41 biopsies remaining for final analysis. There were 29 male (72.5%) and 11 female subjects (27.5%). Mean age was 7.6 ± 3.3 years. Median age was 9 years. Age range was 1–14 years.

The analysis showed that the most common indication of biopsy was nephrotic syndrome in 25 (61%) patients. Other indications were non-nephrotic proteinuria, acute renal failure, chronic renal failure, lupus, and oliguria [Table 1].

**Table 1:** Indications of kidney biopsy

Indication of biopsy	Numbers (%)
Nephrotic syndrome	25 (61)
Proteinuria non-nephrotic	06 (15)
Acute renal failure	03 (07)
Chronic kidney disease	02 (05)
Lupus	03 (07)
Oliguria	01 (02)
Total	41 (100)

Among 25 nephrotic patients, 8 (32%) were steroid dependent and 17 (68%) were steroid resistant. Steroid dependence refers to the relapse on withdrawal of steroids and requirement of steroids to maintain the remission. Steroid resistance refers to little or no reduction in proteinuria after 12–16 weeks of adequate therapy.[5]

The most common histopathology was minimal change disease (MCD) in 15 (37%) patients. It was followed by focal segmental glomerulosclerosis (FSGS) in 5 (12%) patients. Membrano-proliferative glomerulonephritis (MPGN) was noted in 4 (10%) of samples [Table 2].

**Table 2:** Pathological diagnosis of kidney biopsy

Pathological diagnosis	Numbers (%)
Minimal change disease	15 (37)
Focal segmental glomerulosclerosis	05 (12)
Membranoproliferative glomerulonephritis	04 (10)
Membranous nephropathy	03 (07)
Tubulointerstitial nephritis	03 (07)
Renal cortical necrosis	02 (05)
Congenital nephrotic syndrome	02 (05)
Hemolytic uremic syndrome	02 (05)
Crystal nephropathy	01 (02)
Amyloidosis	01 (02)
Mesangial proliferative lupus nephritis	01 (02)
Diffuse proliferative lupus nephritis	01 (02)
Membranous lupus	01 (02)
Total	41 (100)

## Discussion

It is the first report of pediatric renal biopsy from Pakistan. This study provides information about indications of kidney biopsy as well as a pattern of histopathology in children. The most common indication of renal biopsy was nephrotic syndrome. Steroid-resistant nephrotics were twice of steroid-dependent nephrotics in number.

In our study, the most common histopathological diagnosis, irrespective of the indication of biopsy, was MCD. The second most common pathology was FSGS and then MPGN.

Results from some of the representative studies from other geographical locations are presented in Table 3. The three most common histopathologies in NS stand out to be MCD, FSGS, and MPGN, not necessarily in the same order.[6–15] Probably, lower the threshold of doing kidney biopsy, greater the chance of picking up cases of MCD.

**Table 3:** Summary of results of studies from various countries

Ref. No.	Country	Most common indication of renal biopsy	Most common histopathology
6	Italy	NS	MCD
10	Iran	NS	MCD
11	Korea	NS	MCD
12	India	NS	MCD
13	USA	NS	MCD
7	Saudi Arabia	NS	MPGN
8	Turkey	NS	MPGN
9	Croatia	NS	MPGN
14	India	NS	FSGS
15	USA	NS	FSGS

There is ongoing debate regarding the practice patterns of doing pediatric kidney biopsy. Filler and associates form Canada reviewed 17 years data of pediatric renal biopsy.[16] They concluded that in spite of reports of increasing incidence of FSGS, there is no reason to change the initial therapy and current indications to perform renal biopsy in children. Others recommended to modify the current practice of pediatric kidney biopsy to minimize the likelihood of kidney biopsy with MCD.[15] Webb and associates reviewed childhood steroid-sensitive nephrotic syndrome. They concluded that prebiopsy clinical courses do not predict the histological diagnosis.[17]

We recommend to keep low threshold for the procedure, especially because it is now a very safe procedure. Biopsy will help in making early diagnosis and instituting the right doses of steroids or other immunosuppressive medications promptly according to the guidelines published by the Indian Pediatric Nephrology Group.[5]

## Conclusion

Our study is an important contribution to the epidemiology of pediatric renal diseases in South-East Asia. We conclude that MCD is the most common pathology and nephrotic syndrome is the most common indication of biopsy in our children. There is an urgent need of central registry of pediatric kidney biopsy in our country to better understand the pattern of renal disease among children.
